# Supplementary material for: Power of a randomization test in a single case multiple baseline AB design
Source: PLoS One. 2020 Feb 6;15(2):e0228355. doi: 10.1371/journal.pone.0228355 (PMC7004358; doi:10.1371/journal.pone.0228355)
Supplement: S1 File — (DOCX) [file pone.0228355.s001.docx]

**S1 File. Some history of the randomization test.**

Randomization tests trace back to the work of [1] but his conception of an exact test is distinctly different than that of today’s randomization tests. Fisher defined the exact test as a randomization test for which the population is known and so all outcomes are known a priori. Fisher’s randomization tests are statistical tests for interval validity, and the aim was traditionally not to generalize beyond the specific characteristics of the sample. In the single case research this population is most commonly unknown and the data only form a sample of the population. [2-4] was the first to propose a type of randomization test that could be used to draw inferences from a sample without knowing anything about the population. Pittman’s randomization methods was not mechanically different from Fisher’s method and only differed in the conceptualization of what is being inferred. In contemporary social sciences, statistical testing is typically meant to establish external validity, generalizing the results of a sample to an unknown population. In practice, though, the distinction between external and internal validity may not be as critical as stated above [5]. Pure random sampling, a requirement for establishing external validity, is rare and so called convenience sampling is a big threat to the external validity. In addition, one may in some cases generalize the results of a randomization test applied to a small sample to a well thought out population. We think that as with all statistical tests, replication and meta-analysis (see e.g. [6]) are badly needed to assess external validity. In single case designs these replications may even be more important because the number of participants is small, which leads to a larger probability that the sample is not representative for the population.

**References**

1. Fisher, K. (1935). Statistical tests. *Nature,* *136*, 474-474. <https://doi.org/10.1038/136474b0>
2. Pitman, E. (1937). Significance tests which may be applied to samples from any populations. *Supplement to the Journal of the Royal Statistical Society,* *4*, 119-119. <https://doi.org/10.2307/2984124>
3. Pitman, E. (1937). Significance tests which may be applied to samples from any populations. Ii. The correlation coefficient test. *Supplement to the Journal of the Royal Statistical Society,* *4*, 225-225. <https://doi.org/10.2307/2983647>
4. Pitman, E. (1938). Significance tests which may be applied to samples from any populations: Iii. The analysis of variance test. *Biometrika,* *29*, 322-322. <https://doi.org/10.2307/2332008>.
5. Edgington, E. S. (1973). The random-sampling assumption in “comment on component-randomization tests”. *Psychological Bulletin,* *1*, 84-84.
6. Busk, P. L., & Serlin, R. C. (1992). Meta-analysis for single-case research. In T. R. Kratochwill & J. R. Levin (Eds.), *Single-case research design and analysis: New directions for psychology and education* (pp. 187-212). Hillsdale, NJ, US: Lawrence Erlbaum Associates, Inc.

**measurements.**
