# Supplementary material for: Power of a randomization test in a single case multiple baseline AB design
Source: PLoS One. 2020 Feb 6;15(2):e0228355. doi: 10.1371/journal.pone.0228355 (PMC7004358; doi:10.1371/journal.pone.0228355)
Supplement: S2 File — (DOCX) [file pone.0228355.s002.docx]

**S2 File. Explanation of the Koehler and Levin (1998) randomization test.**

Here we show an example of the randomization test elaborated by [1]. First a start moment matrix is defined. In this example there are three possible start moments (*k*) and two participants *(i=1,..,N*) . Note that in Table 1, the possible start moments do not overlap for the two participants.

**Table 1. Start Moment Matrix With Three Possible Start Moments (k) and Two Participants (i).**

|  |  | *k* |  |
| --- | --- | --- | --- |
|  | 1 | 2 | 3 |
| *i= 1* | 3 | 4 | 5 |
| *i= 2* | 6 | 7 | 8 |

When we randomize both the three start moments and the two participants we get $N!\prod_{i=1}^{i=N} k_{i}$ =18 combinations. The second and third columns of Table 2 shows the 18 permutations. From these 18 permutations one is randomly drawn. The data are collected according to the start moments of the intervention of this permutation. For example, we draw permutation 13. In this permutation participant 1 starts the intervention at measurement 6 and participant 2 starts the intervention at measurement 4.

**Table 2. 18 Permutations of Start Moments and Participants.**

| Permutation | Start Intervention | | i=1 | | | i=2 | | | | |  | | |  |
| --- | --- | --- | --- | --- | --- | --- | --- | --- | --- | --- | --- | --- | --- | --- |
| Nr. | i=1 | i=2 | Mean_Ba_ | Mean_Int_ | Mean_Dif_ | Mean_Ba_ | | Mean_Int_ | | Mean_Dif_ | | Average Mean_Dif_ | | |
| 1 | 3 | 6 | 5.00 | 5.63 | -0.63 | 6.40 | 4.75 | | 1.65 | | 0.51 | |  |  |
| 2 | 3 | 7 | 5.00 | 5.63 | -0.63 | 5.83 | 5.33 | | 0.50 | | -0.06 | |  |  |
| 3 | 3 | 8 | 5.00 | 5.63 | -0.63 | 5.43 | 6.50 | | -1.07 | | -0.85 | |  |  |
| 4 | 4 | 6 | 5.67 | 5.43 | 0.24 | 6.40 | 4.75 | | 1.65 | | 0.94 | |  |  |
| 5 | 4 | 7 | 5.67 | 5.43 | 0.24 | 5.83 | 5.33 | | 0.50 | | 0.37 | |  |  |
| 6 | 4 | 8 | 5.67 | 5.43 | 0.24 | 5.43 | 6.50 | | -1.07 | | -0.42 | |  |  |
| 7 | 5 | 6 | 6.25 | 5.00 | 1.25 | 6.40 | 4.75 | | 1.65 | | 1.45 | |  |  |
| 8 | 5 | 7 | 6.25 | 5.00 | 1.25 | 5.83 | 5.33 | | 0.50 | | 0.88 | |  |  |
| 9 | 5 | 8 | 6.25 | 5.00 | 1.25 | 5.43 | 6.50 | | -1.07 | | 0.09 | |  |  |
| 10 | 6 | 3 | 6.00 | 5.00 | 1.00 | 6.50 | 5.43 | | 1.07 | | 1.04 | |  |  |
| 11 | 7 | 3 | 6.17 | 4.50 | 1.67 | 6.50 | 5.43 | | 1.07 | | 1.37 | |  |  |
| 12 | 8 | 3 | 6.00 | 4.33 | 1.67 | 6.50 | 5.43 | | 1.07 | | 1.37 | |  |  |
| 13 | 6 | 4 | 6.00 | 5.00 | 1.00 | 7.00 | 5.00 | | 2.00 | | 1.50 | |  |  |
| 14 | 7 | 4 | 6.17 | 4.50 | 1.67 | 7.00 | 5.00 | | 2.00 | | 1.83 | |  |  |
| 15 | 8 | 4 | 6.00 | 4.33 | 1.67 | 7.00 | 5.00 | | 2.00 | | 1.83 | |  |  |
| 16 | 6 | 5 | 6.00 | 5.00 | 1.00 | 6.25 | 5.20 | | 1.05 | | 1.03 | |  |  |
| 17 | 7 | 5 | 6.17 | 4.50 | 1.67 | 6.25 | 5.20 | | 1.05 | | 1.36 | |  |  |
| 18 | 8 | 5 | 6.00 | 4.33 | 1.67 | 6.25 | 5.20 | | 1.05 | | 1.36 | |  |  |

dark shaded row: de permutation that is drawn for the data collection. Light shaded rows, permutations that have averaged mean differences equal or larger than the observed mean difference.

Table 3 shows an example of the observed scores for the two participants in the baseline and the intervention phase. Note that there were 10 measurements for participant 1and 9 measurements for participant 2. In a multiple baseline design the number of measurements are allowed to differ for participants.

**Table 3. Example of the Observed Scores for the Two Participants in the Baseline and the Intervention Phase.**

| Measurement | 1 | 2 | 3 | 4 | 5 | 6 | 7 | 8 | 9 | 10 |
| --- | --- | --- | --- | --- | --- | --- | --- | --- | --- | --- |
| i=1 | Baseline | | | | | Intervention | | | | |
| Score | 4 | 6 | 7 | 8 | 5 | 7 | 5 | 5 | 3 | 5 |
| i=2 | Baseline | | | Intervention | | | | | | |
| Score | 6 | 7 | 8 | 4 | 7 | 3 | 3 | 6 | 7 |  |

The mean baseline scores are 6 and 7 for participants 1 and 2 and the mean intervention scores are 5 for both participants. The mean difference score for participant 1 is 6-5=1 and for participant 2: 7-5=2. The average mean difference over participants is (2+1)/2=1.5. This is the observed test statistic.

To calculate the permutation distribution of the mean difference the average mean differences are calculated for all other permutations. These mean differences are shown in columns 4 through 9 in Table 2. Table 4 shows how the mean differences are calculated for permutation 1. In permutation 1 the intervention starts at measurement 3 for participant 1 and at measurement 6 for participant 2.

**Table 4. Example of the Scores for the Two Participants in the Baseline and the Intervention Phase for Permutation 1.**

| Measurement | 1 | 2 | 3 | | 4 | | 5 | | 6 | 7 | 8 | 9 | 10 |
| --- | --- | --- | --- | --- | --- | --- | --- | --- | --- | --- | --- | --- | --- |
| i=1 | Baseline | |  |  | |  | | Intervention | | | | | |
|  | 4 | 6 | 7 | | 8 | | 5 | | 7 | 5 | 5 | 3 | 5 |
| i=2 | Baseline | | | | | | | | Intervention | | | | |
|  | 6 | 7 | 8 | | 4 | | 7 | | 3 | 3 | 6 | 7 |  |

The mean baseline scores are 5 and 6.4 for participants 1 and 2 and the mean intervention scores are 5.63 and 4.75 respectively. The mean difference score for participant 1 is 5-5.63=-0.63 and for participant 2: 6.4-4.75=1.65. The average mean difference over participants is (-0.63+1.65)/2 = 0.51.

Finally we can calculate the *p*-value by counting the number of times the average mean difference is equal or larger than the observed mean difference, divided by the total number of permutations. In our example there are three permutations (including the permutation that was drawn for observing the scores) that have an average mean difference of 1.5 or larger. Our *p*-value is thus: 3/18=.167. Note that since we only have 18 permutations our *p*-value cannot reach statistical significance (when alpha = .05).

Fig 1 shows the density plot of the 18 permutations. Note that this distribution is not symmetrical and the mean of the mean differences is not 0.


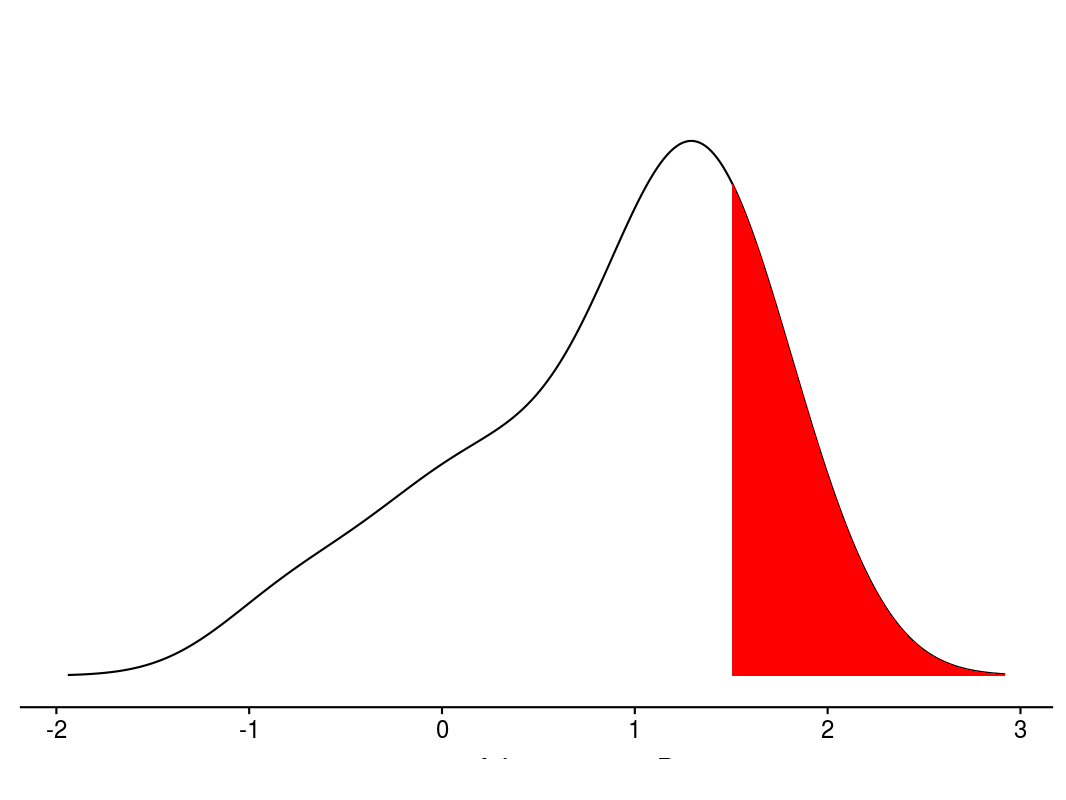


**Fig 1. Density plot of the mean differences of the 18 permutations. Shaded part shows the probability of the observed mean difference or a larger one.**

**References**

1. Koehler, M. J., & Levin, J. R. (1998). Regulated randomization: A potentially sharper analytical tool for the multiple-baseline design. *Psychological Methods*, *3*, 206-217.
