# Supplementary material for: Power of a randomization test in a single case multiple baseline AB design
Source: PLoS One. 2020 Feb 6;15(2):e0228355. doi: 10.1371/journal.pone.0228355 (PMC7004358; doi:10.1371/journal.pone.0228355)
Supplement: S3 File — (DOCX) [file pone.0228355.s003.docx]

**S3 File. R Code of the Function Used in the Simulation Study**

Below the R code is shown for the function that was used to calculate the power in a given design. The actual code is in courier font, the explanation is introduced by a hashtag and in times new roman font.

MBD.P<-function(nr.pp,nr.st,BT.r,Eq.obs.in.BT,NonOverlap.st,nr.meas, mean.dif,sd.B,sd.I,AR,alpha,nr.samples,nr.rep.pow){

# nr.pp: number of participants ranges from 2 to 12.

# nr.st: number of possible start moments per participant, values: 2,3,4.

# BT.r: correlation between mean baseline and mean intervention observations, values: 0,.7.

# Eq.obs.in.BT: equal number of observations in baseline and intervention phase, values: TRUE, FALSE.

# NonOverlap.st: possible start moments per participant are unique (not-overlapping): TRUE, FALSE.

# nr.meas: number of measurements in baseline and intervention together, values: 15, 30, 60.

# mean.dif: difference in mean in baseline and intervention phase, values: .3, .6, 1.

# sd.B: standard deviation within participant of the baseline observations, values: .67,1,1.33.

# sd,I: standard deviation within participant of the intervention observations, values: .67,1,1.33.

# AR: autocorrelation of scores within participants, values: 0, 0.1, 0.2, 0.3, 0.4, 0.5.

# alpha: nominal type 1 error. Value=.05.

# nr.samples: number of samples on which a randomization test is performed. Value=500.

# nr.rep.pow: number of replications to calculate the power. Value=100.

### Starttable ####

# We start by creating the "starttable". This table is a matrix with nr.pp rows and nr.st columns. The rows contain the possible start moments of the intervention per participant. The possible start moments may overlap (Overlap.st=FALSE) or not (Overlap.st=TRUE). The range of possible start moments may be at the center of all measurements (Eq.obs.in.BT=TRUE) or not (Eq.obs.in.BT==FALSE). At least two observations are required in both the Baseline and the intervention phase.

if(NonOverlap.st==TRUE){

if(Eq.obs.in.BT==TRUE){start=round(median(c(1:nr.meas))-nr.st*nr.pp*.5)

if(start<3){start=3}

}else{start=3}

#Eq.obs.in.BT==FALSE

starttable<-t(matrix(seq(start,nr.st*nr.pp+start-1),nr.st,nr.pp))

}else{

#NonOverlap.st=FALSE

if(Eq.obs.in.BT==TRUE){start=round(median(c(1:nr.meas))-(nr.st+nr.pp-1)*.5)

}else{start=3}

#Eq.obs.in.BT==FALSE

starttable<-matrix(NA,nr.pp,nr.st)

for(i in c(1:nr.pp)){

starttable[i,seq(nr.st)]<-seq(start+(i-1),start+(i-1)+nr.st-1)

}

}

### Creating assignment matrix ####

# Next a matrix is created that contains all possible assignments of start moments to participants. This matrix is called the "ass.table" . In total the number of possible assignments is: nr.pp!*nr.st^nr.pp. Because this number becomes very large when the number of participants is larger than 5, we created the full assignment matrix only when nr.pp is smaller than 6. From nr.pp>5, a sample of 400 assignments is drawn from all possible assignments.

if(nr.pp<6){

all.perm<-matrix(NA,nr.st^nr.pp,nr.pp)

v=nr.st^(nr.pp-1)

p=1

for(i in c(1:nr.pp)){

all.perm[,i]<-rep(sort(rep(c(1:nr.st),v)),p)

p=nr.st*p

v=v/nr.st

}

perm.values<-matrix(NA,nr.st^nr.pp,nr.pp)

for(k in c(1:nr.pp)){

t=1

for(i in all.perm[,k]){

perm.values[t,k]<-starttable[k,i]

t=t+1

}

}

}

time<-system.time({

result<-foreach(sim =1:nr.rep.pow,.combine=cbind) %dopar% {

if(nr.pp<6){

ass.table<-matrix(NA,factorial(nr.pp)*nrow(perm.values),nr.pp)

#all permutations

for(i in c(1:nrow(perm.values))){

ass.table[c((i*factorial(nr.pp)-(factorial(nr.pp)-1)):(i*factorial(nr.pp))),c(1:nr.pp)]<-permutations(nr.pp,nr.pp,perm.values[i,],set=FALSE)

}

if(nrow(ass.table)>400){ass.table<-ass.table[sample(nrow(ass.table), 400), ]}

}else{

ass.table<-matrix(NA,400,nr.pp)

for(j in seq.int(400)){

x<-sample(c(1:nr.pp),nr.pp)

for(i in c(1:nr.pp)){

ass.table[j,i]<-sample(starttable[x[i],],1)

}

}

}

### Creating observations in baseline and intervention phase ####

#for power en type 1

p.value<-matrix(NA,nr.samples,2)

#for effect size

obs.es<-matrix(NA,nr.samples,nr.pp)

for(q in seq.int(nr.samples)){

# sample.assign is a randomly drawn row from the assignment matrix. In a real world application of the randomization test this sample.assign contains the start moment of the intervention for each participant.

sample.assign<-sample(seq.int(nrow(ass.table)),1)

obs.power<-matrix(NA,ncol(ass.table),nr.meas)

obs.type1<-matrix(NA,ncol(ass.table),nr.meas)

for(j in seq.int(ncol(ass.table))){

# the error for the scores in the baseline/intervention scores are drawn from a normally distribution with mean 0 and sd.B/sd,I. The number of scores that are drawn is determined by the start moment (from sample.assign) of the intervention.

e.B<-rnorm(ass.table[sample.assign,j]-1,0,sd.B)

e.I<-rnorm(nr.meas-length(e.B),0,sd,I)

#vector of scores Baseline phase

baseline<-rep(NA,length(e.B)).

#vector of scores intervention phase

intervention<-rep(NA,length(e.I))

#vector of scores intervention phase with no effect (for type 1 error rate)

intervention.t1<-rep(NA,length(e.I))

# The first observation in the baseline/intervention phase is equal to the first error score.

baseline[1]<-e.B[1]

intervention[1]<-e.I[1]+mean.dif

intervention.t1[1]<-e.I[1]

for(i in c(2:(ass.table[sample.assign,j]-1))){

# the remaining baseline scores are created by a autoregressive function: autocorrelation (AR) times the previous baseline score (t-1) plus the error at t.

baseline[i]<-AR*baseline[i-1]+e.B[i]

}

#no correlation between baseline and intervention means within a participant

if(BI.r==0){

for(i in c(2:length(e.I))){

# the remaining intervention scores are created by a autoregressive function: autocorrelation (AR) times the previous intervention score (t-1), plus the mean difference, plus the error at t. For the type 1 error the mean.dif = 0.

intervention[i]<-AR*e.I[i-1]+mean.dif+e.I[i]

intervention.t1[i]<-AR*e.I[i-1]+e.I[i]

}

}else{

# mean baseline score + mean diff, is mean in intervention within a subject

if(BI.r==1){

for(i in c(2:length(e.I))){

intervention[i]<-

mean(baseline)+AR*e.I[i-1]+mean.dif+e.I[i]

intervention.t1[i]<-

mean(baseline)+AR*e.I.t1[i-1]+e.I.t1[i]

}

}

}

### Calculating means for baseline and interventions scores for all rows in the assignment table ####

#combining baseline and intervention scores

obs.power[j,seq.int(nr.meas)]<-c( baseline, intervention)

#combining baseline and intervention (mean.dif=0) scores

obs.type1[j,seq.int(nr.meas)]<-c( baseline, intervention.t1)

# For each sample q, for each participant j, the within participant effect size is calculated by (mean baseline) minus (mean intervention) divided by the pooled standard deviation.

obs.es[q,j]<-mean(intervention)-(mean(baseline))/(

(sd(baseline)*(ass.table[sample.assign,j]-1) +sd(intervention)*(nr.meas-ass.table[sample.assign,j]-1))/nr.meas)

}

#table containing the mean baseline and mean intervention score for each participant for each row in the assignment table

df.power<-matrix(NA,nrow(ass.table),ncol(ass.table)*2)

#table containing the mean baseline and mean intervention score (mean.dif=0) for each participant for each rwo in the assignment table

df.type1<-matrix(NA,nrow(ass.table),ncol(ass.table)*2)

for(j in seq.int(ncol(ass.table))){

for(i in seq.int(nrow(ass.table))){

df.power[i,c(2*j-1,2*j)]<-c(mean(obs.power[j,seq.int(ass.table[i,j]-1)]), mean(obs.power[j,c(ass.table[i,j]:nr.meas)]))

df.type1[i,c(2*j-1,2*j)]<-c(mean(obs.type1[j,seq.int(ass.table[i,j]-1)]), mean(obs.type1[j,c(ass.table[i,j]:nr.meas)]))

}

}

# table containing the difference in means between the baseline and intervention score for each participant, for each row in the assignment table

mean.dif.BI.power<-matrix(NA,nrow(ass.table),ncol(ass.table))

mean.dif.BI.type1<-matrix(NA,nrow(ass.table),ncol(ass.table))

for(i in seq(1,ncol(df.power),by=2)){

mean.dif.BI.power[,(i+1)/2]<-df.power[,i]-df.power[,i+1]

mean.dif.BI.type1[,(i+1)/2]<-df.type1[,i]-df.type1[,i+1]

}

# vector containing the average mean difference over participant for each row in the assignment table

power.means<-apply(mean.dif.BI.power,1,mean)

type1.means<-apply(mean.dif.BI.type1,1,mean)

# the p value for each sample q is calculated by counting the number of times the average difference in means is equal or larger than the average mean difference of the drawn assignment of start moments (sample.assign)

p.value[q,1] <-length(power.means[power.means<=power.means[sample.assign]])/ length(power.means)

p.value[q,2] <- length(type1.means[ type1.means<= type1.means[sample.assign]])/length(type1.means)

}

# the power is calculated by counting the number of samples that has a p-value smaller than alpha.

power<-length(p.value[p.value[,1]<alpha,1])/length(p.value[,1])

# the actual type 1 error culated by counting the number of samples that has a p-value smaller than alpha.

type1<-length(p.value[p.value[,2]<alpha,2])/length(p.value[,2])

# the mean effect size is calculated by averaging over all effect sizes (over participants and over samples)

mean.obs.es<-mean(rowMeans(obs.es))

end<-list(power,type1,mean.obs.es)

end

}

})

res<-t(matrix(result,3,nr.rep.pow))

return(res)

}
