# Supplementary material for: Power of a randomization test in a single case multiple baseline AB design
Source: PLoS One. 2020 Feb 6;15(2):e0228355. doi: 10.1371/journal.pone.0228355 (PMC7004358; doi:10.1371/journal.pone.0228355)
Supplement: S4 File — (DOCX) [file pone.0228355.s004.docx]

**S4 File. Autocorrelation and power in multiple baseline across subjects designs.**

Since the observations in single case designs are time series there is a dependency between the observations within a participant and the observations may well be autocorrelated. [1] show that the degree of positive autocorrelation in multiple baseline designs is negatively related to power. In our study, we replicated this finding and we additionally want to evaluate whether the effect of autocorrelation on the power of the randomization test is influenced by the seven design factors we took into account. Because we had no a priori expectations that there would be an interaction effect of degree of positive autocorrelation on the power we did not fully cross all factor levels but started by formulating a default design (see Table 2, main text), which has 60 measurements, three possible start moments, no overlap in possible start moments, an equal number of measurements in the baseline and the intervention phase, standard deviations of 1 in both the baseline and the intervention phase, a mean difference of 1 between the baseline and the intervention observations, and no correlated means of the baseline and the intervention phase. Next we formulated nine alternative designs in which only one of the factor levels was different from the default design. For these ten designs we simulated data in the same way as was done for the simulations in the main text with autocorrelations 0, .1, .2, .3, .4 and .5 with both four and eight participants. For these 10 (designs) *6 (autocorrelations)*2 (# participants) = 120 combinations the power was calculated.

Fig 1 (main text) shows that there is an effect of autocorrelation on the power. This effect is shown for all of the ten designs and data simulated both with four and eight participants. The interaction effect of the other design factors and autocorrelation, however, did not seem to exist since the regression lines of the 10 situations are rather parallel except for some ceiling effects. The regression lines in Fig 1 seem to indicate that the power of a design with autocorrelated data can be predicted by the power of that design with uncorrelated data (autocorrelation = 0) and the standard deviation of the power of that design with uncorrelated data. When this would indeed be the case, this would be useful for predicting the power of a multiple baseline design in practice, because one only needs the power and standard deviation of the power when autocorrelation is zero for a particular design, instead of simulating data for all possible autocorrelations for every possible design.

In order to evaluate this idea we followed the following steps:

1. For all of the 20 designs described above we did a regression analysis in which the power was predicted by the autocorrelation. We saved the 20 *slopes* resulting from these analyses (Table 6, 5^th^ column).

**Table 1. Power and Standard Deviation of the Power when Autocorrelation was 0 and Slope of the Regression Line for the 20 Situations.**

| Design | # pp | Power AR=0 | SD Power AR=0 | Slope |
| --- | --- | --- | --- | --- |
| default | 4 | 0.747 | 0.018 | -0.495 |
| nr.st=2 | 4 | 0.614 | 0.022 | -0.346 |
| nr.st=4 | 4 | 0.839 | 0.018 | -0.562 |
| BI.r=0.7 | 4 | 0.759 | 0.017 | -0.501 |
| Eq.obs.in.BI=FALSE | 4 | 0.495 | 0.023 | -0.239 |
| NonOverlap.st=FALSE | 4 | 0.451 | 0.020 | -0.182 |
| Mean.dif=.3 | 4 | 0.170 | 0.016 | -0.061 |
| Mean.dif=.6 | 4 | 0.406 | 0.021 | -0.266 |
| sd.B=2*sd.I | 4 | 0.720 | 0.021 | -0.518 |
| sd.T=2*sd.B | 4 | 0.722 | 0.021 | -0.436 |
| default | 8 | 0.991 | 0.005 | -0.323 |
| nr.st=2 | 8 | 1.000 | 0.001 | -0.121 |
| nr.st=4 | 8 | 1.000 | 0.001 | -0.140 |
| BI.r=0.7 | 8 | 0.950 | 0.011 | -0.418 |
| Eq.obs.in.BI=FALSE | 8 | 0.916 | 0.014 | -0.480 |
| NonOverlap.st=FALSE | 8 | 0.999 | 0.001 | -0.192 |
| Mean.dif=.3 | 8 | 0.409 | 0.019 | -0.358 |
| Mean.dif=.6 | 8 | 0.886 | 0.014 | -0.676 |
| sd.B=2*sd.I | 8 | 0.998 | 0.002 | -0.320 |
| sd.I=2*sd.B | 8 | 0.998 | 0.002 | -0.165 |

1. Next we predicted the 20 *slopes* resulting from step 1 by the power of the design when autocorrelation was 0 (Table 6, column 3), the standard deviation of the power of the design when autocorrelation was 0 (Table 6, column 4) and the interaction (i.e. product) of these two predictors. This analysis resulted in an intercept, *b_0_* *=-.662*, a regression weight for the power when autocorrelation was 0, *b_1_=.512*, a regression weight for the standard deviation of the power when autocorrelation was 0, *b_2_=41.404*, and a regression weight for the interaction between the power when autocorrelation was 0 and the standard deviation of the power when autocorrelation was 0, *b_3_=-71.510*.
2. The power for the of autocorrelations .1, .2, .3, .4, and .5 (*AR = .1,..,.5*) for the 20 situations (*s = 1,…,20*) was then predicted by:

power_AR,s_ = power _AR=0,s_ + (-.662+0.512*power _AR=0,s_+41.404* SDpower _AR=0,s_ + -71.510* power _AR=0,s_* SDpower _AR=0,s_)*AR.

This resulted in 100 predicted powers.

1. Next, these 100 predicted powers were compared with the simulated powers using again a linear regression. The *R^2^* was .992 indicating that the power when autocorrelation is not 0 could almost perfectly by predicted by the power and the standard deviation when autocorrelation is 0.
2. In the previous steps the same data was used to predict the power and to calculate the regression coefficients *b_0_, b_1_, b_2,_* and *b_3_* from step 2. This could be a reason to the almost perfect fit. We therefore evaluated whether the same coefficients (*b_0_, b_1_, b_2,_* and *b_3_*) could also predict the power of new simulated data for the 10 design and AR =0, .1.,.2,.3, .4, .5 when the number of participants was six.

The *R^2^* for predicting the simulated power by the power and standard deviation when the autocorrelation was 0, was 0.985 indicating that the regression coefficients obtained from simulations with *pp=4* and *pp=8* could very well predict the power for the 10 situations when the number of participants was 6.

1. As a final check we randomly draw 100 situations from the 624 situations (see main text) for which the power was simulated having autocorrelation is 0. For each of these situations we randomly draw one of the five autocorrelations (.1-.5) and calculated the power for these situations. Next we compared the power resulting from the simulated data for these situations with the predicted power for these situations by the regression formula described above, with the regression coefficients having the same values. The *R^2^* was .989. Fig 2 shows a scatterplot of the power of the 100 samples predicted by the power when the autocorrelation was 0 and the standard deviation of the power when the autocorrelation was 0.


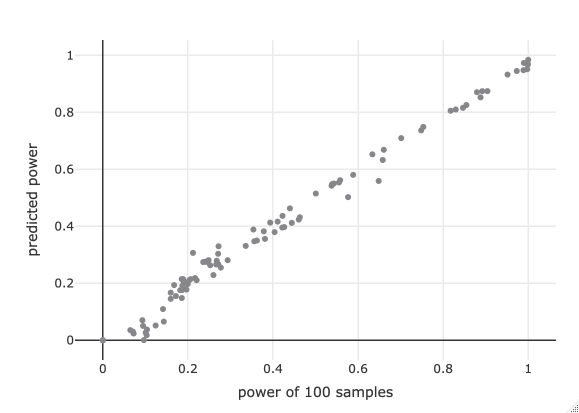


**Fig 1. Scatterplot of the power of the 100 random drawn designs predicted by the power and the standard deviation of the power when the autocorrelation was 0.**

**References**

1. Ferron, J., & Sentovich, C. (2002). Statistical power of randomization tests used with multiple-baseline designs. *Journal of Experimental Education, 70*, 165-178. <https://doi.org/10.1080/00220970209599504>
