# Supplementary material for: Power of a randomization test in a single case multiple baseline AB design
Source: PLoS One. 2020 Feb 6;15(2):e0228355. doi: 10.1371/journal.pone.0228355 (PMC7004358; doi:10.1371/journal.pone.0228355)
Supplement: S5 File — (DOCX) [file pone.0228355.s005.docx]

**S5 File. Correcting for Gradually emerging patterns.**

Most of the times researchers will be interested in the final effect of the intervention and not in the average effect that includes the emerging effect measurements. In this case one may prefer to exclude the measurements in which the effect was not fully emerged from the randomization test. Although one may not know exactly how many interventions measurements have to be excluded, there may be some valid information from the literature or from previous experience. In order to evaluate the power of the randomization test when a number of intervention measurements in which the effect is assumed to be emerging is removed we did another simulation study. In this study we simulated a gradually emerging effect that developed during the first 10 intervention measurements. Next, we calculated the power of the randomization test by varying the user defined number of measurements that the intervention effect was assumed to fully develop from 1 (suddenly emerging effect) to 15. The other factor levels were set to the default (see Table 4 main text). Fig. 1 shows the estimated power for the 15 situations. This figure shows that when number of measurements that the effect is assumed to be developing is much underestimated, the effect on the power is large. When there is a small underestimation of maximally three measurements the effect on the power is small. An overestimation of the number of measurements that the intervention effect is assumed to be emerging does not influence the power.


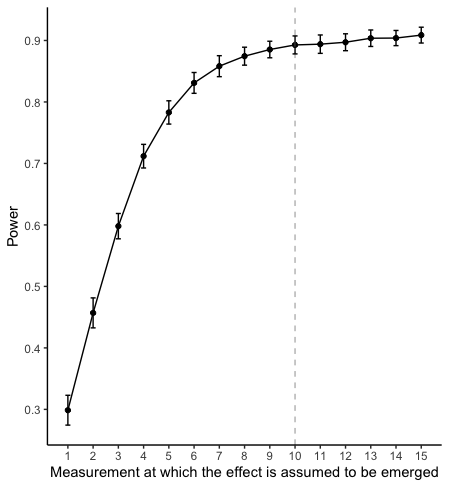


**Fig 1. Estimated power when the effect is emerging during 10 (dashed horizontal line) intervention measurements and the randomization excludes 1 to 15 intervention**
