# Supplementary material for: Power of a randomization test in a single case multiple baseline AB design
Source: PLoS One. 2020 Feb 6;15(2):e0228355. doi: 10.1371/journal.pone.0228355 (PMC7004358; doi:10.1371/journal.pone.0228355)
Supplement: S1 Table — (DOCX) [file pone.0228355.s006.docx]

**S1 Table. Overview of Possible (grey) and Impossible (black) Combinations of Number of Participants, Number of Start Moments (k), Number of Measurements (t) and Unique Start Moments or Overlap in Start Moments.**

|  |  |  | Number of Participants | | | | | | | | | | |
| --- | --- | --- | --- | --- | --- | --- | --- | --- | --- | --- | --- | --- | --- |
|  | *t* | *k* | 2 | 3 | 4 | 5 | 6 | 7 | 8 | 9 | 10 | 11 | 12 |
| Unique | 15 | 2 |  |  |  |  |  |  |  |  |  |  |  |
|  |  | 3 |  |  |  |  |  |  |  |  |  |  |  |
|  |  | 4 |  |  |  |  |  |  |  |  |  |  |  |
| Overlap | 15 | 2 |  |  |  |  |  |  |  |  |  |  |  |
|  |  | 3 |  |  |  |  |  |  |  |  |  |  |  |
|  |  | 4 |  |  |  |  |  |  |  |  |  |  |  |
| Unique | 30 | 2 |  |  |  |  |  |  |  |  |  |  |  |
|  |  | 3 |  |  |  |  |  |  |  |  |  |  |  |
|  |  | 4 |  |  |  |  |  |  |  |  |  |  |  |
| Overlap | 30 | 2 |  |  |  |  |  |  |  |  |  |  |  |
|  |  | 3 |  |  |  |  |  |  |  |  |  |  |  |
|  |  | 4 |  |  |  |  |  |  |  |  |  |  |  |
