# Supplementary material for: Power of a randomization test in a single case multiple baseline AB design
Source: PLoS One. 2020 Feb 6;15(2):e0228355. doi: 10.1371/journal.pone.0228355 (PMC7004358; doi:10.1371/journal.pone.0228355)
Supplement: S2 Table — (DOCX) [file pone.0228355.s007.docx]

**S2 Table. Mean and SD of the Type I Error for All Factor Levels**

| Factor | *Mean* | | | *SD* | |  | | |
| --- | --- | --- | --- | --- | --- | --- | --- | --- |
| \| *Number of participants* \|  \| \| --- \| --- \| | | | | |  |  |  |  |
| 2 | 0.010 | | | | 0.003 | |  |  |
| 3 | 0.046 | | | | 0.009 | |  |  |
| 4 | 0.049 | | | | 0.010 | |  |  |
| 5 | 0.048 | | | | 0.010 | |  |  |
| 6 | 0.050 | | | | 0.010 | |  |  |
| 7 | 0.050 | | | | 0.010 | |  |  |
| 8 | 0.050 | | | | 0.010 | |  |  |
| 9 | 0.050 | | | | 0.010 | |  |  |
| 10 | 0.050 | | | | 0.010 | |  |  |
| 11 | 0.050 | | | | 0.010 | |  |  |
| 12 | 0.050 | | | | 0.010 | |  |  |
| *Number of start moments intervention* | | |  |  |  |  |  |  |
| 2 | 0.050 | | | | 0.010 | | |  |
| 3 | 0.050 | | | | 0.010 | | |  |
| 4 | 0.050 | | | | 0.010 | | |  |
| *Effect size* |  | | | |  | | |  |
| 0.3 | 0.050 | | | | 0.010 | | |  |
| 0.6 | 0.050 | | | | 0.010 | | |  |
| 1 | 0.050 | | | | 0.010 | | |  |
| *Equal Number of Measurements Baseline & Intervention* | |  |  |  |  |  |  |  |
| TRUE | 0.050 | | | | 0.010 | | |  |
| FALSE | 0.050 | | | | 0.010 | | |  |
| *Non-overlap of start moments intervention* |  | | | |  | | |  |
| TRUE | 0.050 | | | | 0.010 | | |  |
| FALSE | 0.050 | | | | 0.010 | | |  |
| *Correlated baseline and intervention observations* | | | | | | | |  |
| TRUE | 0.050 | | | | 0.010 | | |  |
| FALSE | 0.050 | | | | 0.010 | | |  |
| *Ratio Standard deviation (s) in baseline (b) and intervention (i) phase* | | | | | | | |  |
| 2s_b_ = s_i_ | 0.051 | | | | 0.010 | | |  |
| s_b_ = s_i_; | 0.049 | | | | 0.010 | | |  |
| s_b_ = 2s_i_ | 0.049 | | | | 0.010 | | |  |
| *Number of measurements* | | | | |  | | |  |
| 15 | 0.049 | | | | 0.010 | | |  |
| 30 | 0.050 | | | | 0.010 | | |  |
| 60 | 0.050 | | | | 0.010 | | |  |
